# Mechanosensitive miRNAs and Bone Formation

**DOI:** 10.3390/ijms18081684

**Published:** 2017-08-02

**Authors:** Zhihao Chen, Yan Zhang, Chao Liang, Lei Chen, Ge Zhang, Airong Qian

**Affiliations:** 1 Laboratory for Bone Metabolism, Key Laboratory for Space Bioscience and Biotechnology, School of Life Sciences, Northwestern Polytechnical University, Xi’an 710072, China; chzhh@mail.nwpu.edu.cn (Z.C.); zhangyan0911@mail.nwpu.edu.cn (Y.Z.); chenlei1994@mail.nwpu.edu.cn (L.C.); 2 NPU-HKBU Joint Research Centre for Translational Medicine on Musculoskeletal Health in Space, Northwestern Polytechnical University, Xi’an 710072, China; liangchao512@163.com; 3 Shenzhen Research Institute of Northwestern Polytechnical University, Shenzhen 518057, China; 4 Institute for Advancing Translational Medicine in Bone and Joint Diseases, School of Chinese Medicine, Hong Kong Baptist University, Hong Kong 999077, China

**Keywords:** mechanical stimuli, mechanosensitive miRNAs, bone formation, osteogenic differentiation

## Abstract

Mechanical stimuli are required for the maintenance of skeletal integrity and bone mass. An increasing amount of evidence indicates that multiple regulators (e.g., hormone, cytoskeleton proteins and signaling pathways) are involved in the mechanical stimuli modulating the activities of osteogenic cells and the process of bone formation. Significantly, recent studies have showed that several microRNAs (miRNAs) were sensitive to various mechanical stimuli and played a crucial role in osteogenic differentiation and bone formation. However, the functional roles and further mechanisms of mechanosensitive miRNAs in bone formation are not yet completely understood. This review highlights the roles of mechanosensitive miRNAs in osteogenic differentiation and bone formation and underlines their potential therapeutic application for bone loss induced by the altering of mechanical stimuli.

## 1. Introduction

Bone, as a specifically mechanical sensory organ, is constantly remodeled (bone formation and bone resorption) under a normal mechanical environment [1,2]. Post-natal bone formation is controlled by osteogenic cells, which are believed to be responsive to different mechanical stimuli [3,4]. Clinically, a lack of mechanical stimuli (e.g., aging, paralysis) or exposure to external mechanical unloading (e.g., during bed-rest or hind-limb unloading, or under the conditions experienced by astronauts) reduce weight-bearing bone formation and weaken bone structure [5,6,7,8]. Previous reports from our laboratory and others have demonstrated that multifactorial regulators are involved in the mechanical stimuli modulating the activity of osteogenic cells and bone formation, such as hormones or cytokine (e.g., parathyroid hormone [9], glucocorticoid [10], follistatin-like 3 [11]), cytoskeleton proteins (e.g., microtubule actin crosslinking factor 1 [12], connexin 43 [13]), signaling pathways (e.g., Wnt signaling [14,15]) and microRNAs (miRNAs).

miRNAs, a newly discovered class of evolutionarily conserved short noncoding RNAs, play a vital role in diverse physiological and pathological processes, including cell differentiation, proliferation, apoptosis and cancer development [16,17,18]. Emerging evidence has showed that miRNAs are involved in bone formation [19,20,21]. Significantly, recent studies have discovered that miR-503-5p [22] and miR-103a [23] are sensitive to different mechanical stimuli when regulating osteogenic cell differentiation and bone formation. Here, miRNA, which expression alters with mechanical use or disuse, was regarded as “mechanosensitive microRNA”. However, the cellular and molecular mechanisms underlying how mechanosensitive miRNAs impacted on bone formation have not yet been completely understood. This review aims to provide an overview of the relationship between mechanosensitive miRNAs and bone formation, including mechanosensitive miRNAs and osteogenic differentiation; mechanosensitive miRNAs and osteoblast proliferation; and mechanosensitive miRNAs and bone formation.

## 2. Biogenesis and Function of miRNAs

miRNAs are evolutionary conserved short (~22 nucleotides) non-coding single-stranded RNAs, that emerged as post-transcriptional regulators of gene expression either by repressing target messenger RNAs (mRNAs) translation efficiency or resulting in target mRNAs degradation [17,24,25]. Mature miRNAs are produced through a series of biological processes which are initiated with transcription [26]. The primary transcript (pri-miRNA), hundreds of nucleotides long, has a local hairpin structure embedding mature miRNA sequences, and pri-miRNA is transcribed from the intragenic or intergenic DNA regions carried out by RNA polymerase II. Following transcription, pri-miRNA is cleaved by Drosha or DGCR8 (Di George syndrome critical gene 8) into precursor miRNA (pre-miRNA) with a ~70 nucleotide stem-loop structure. Then, pre-miRNA is exported from the nucleus to the cytoplasm by the exportin-5 complex and processed to form mature miRNA by Dicer in the cytoplasm. Finally, mature miRNA is assembled into an RNA-induced silencing complex (RISC) with Ago 2 (Argonaute 2), which guides RISC directly to the UTR (untranslated regions) of its target mRNA [26,27].

It has been reported that multiple miRNAs have been postulated to play a vital role in diverse biological processes, including bone formation [21,27]. The importance of miRNAs in bone formation has been addressed by the osteoblast-type-specific deletion of Dicer, required for miRNA biogenesis, generating with a perinatal phenotype of delayed-bone mineralization and a subsequent increase in postnatal bone formation [28]. Additionally, Dicer deficiency in osteoclasts not only reduced the number of osteoclasts and the osteoclast surface, but also suppressed the mineral apposition rate (MAR) and bone formation rate (BFR) in vivo [29]. There is an increasing amount of evidence that miRNAs are also involved in the cellular response to various mechanical stimuli in diverse organs and cells [30,31,32,33,34,35]. Significantly, recent studies have discovered that some mechanosensitive miRNAs played important roles in the differentiation or proliferation of osteogenic cells in vitro and bone formation in vivo [22,23,36,37,38,39,40].

## 3. Mechanosensitive miRNAs and Osteogenic Differentiation

Mechanosensitive miRNAs are identified to be involved in osteogenic differentiation due to the expressions of these miRNAs changing with mechanical stimuli, altering osteogenic differentiation. Microarray and bioinformatics analysis are effective and prompt methods to search for mechanosensitive miRNAs altering with different mechanical stimuli in osteogenic cells (Table 1). Two microarray data of MC3T3-E1 cells subjected to a four-point bending stretch suggested that miR-3077-5p, -3090-5p, -3103-5p, -191*, and -3070a were significantly up-regulated, in contrast, miR-466i-3p, -466h-3p, -218 and -33 were down-regulated; accompanied by alkaline phosphatase (ALP) activity, mRNA levels of *Alp*, *Ocn* (osteocalcin), *Col I* (Collage I), and the protein levels of BMP-2 and BMP-4 increased in the mechanical stretch group [41,42]. Furthermore, analysis of putative target genes indicated that this mechanosensitive miRNAs might be involved in osteoblast differentiation [41,42]. Additionally, four-point bending stretch could have significantly promoted cementoblastogenesis, mineralized nodule formation, and miR-146b-5p expression in OCCM-30 cells. *Smad4* (SMAD family member 4), a common mediator of both BMP (Bone morphogenetic protein) and TGF-β (Transforming growth factor-β) signaling, was a target gene of mechanosensitive miR-146b-5p; this was confirmed by a dual-luciferase reporter assay [43].

According to Wolff’s law, mechanical forces are divided into stretch stress, hydrostatic compressive force, and shear stress due to fluid flow in vitro [44]. Four-point bending and Flexcell were both used to supply stretch stress in osteogenic cells. The Flexcell stretch system was a common device for studying the effects of mechanical stretch on periodontal ligament stem cells (PDLSCs) and periodontal ligament cells (PDLCs), which were capable of osteogenic differentiation under mechanical stimuli [39,45]. In genome-wide miRNA expression profiling of PDLSCs, the expressions of miR-1246, -5096, -638, -663, -21, -4492, and -4734 increased, while miR-3195, -4281, and -3178 decreased under Flexcell stretch [46]. Another microarray indicated that the expressions of miR-195-5p, -424-5p, -1297, -3607-5p, -145-5p, -4328, and -224-5p were significantly lower in PDLCs subjected to Flexcell stretch for 72 h [47]. Fluid shear stress (FSS), another common and potent type of mechanical stimulus in bone cells, enhanced osteogenic gene expression, ALP activity, and mineralization, accompanied with a decrease of miR-20a, -21, -19b, -34a, -34c, -140, and -200b in MC3T3-E1 cells [48]. The above-mentioned mechanosensitive miRNAs were putatively involved in osteogenic differentiation (Table 1). In our study, a biaxial random positioning machine (RPM) was used to investigate the mechanical unloading effects on osteoblasts [49] and osteocytes [50]. In RPM microarray data [50], we found that miR-15a (fold change = 0.426), miR-221(fold change = 0.665) and miR-29a (fold change = 0.513) were down-regulated in MLO-Y4 osteocytes under an RPM unloading condition [50].

### 3.1. Mechanosensitive miRNAs Promoted Osteogenic Differentiation

Several mechanosensitive miRNAs were reported to promote osteogenic differentiation (Table 2) [39,40]. To deeply explore the function of mechanosensitive miR-21 on the osteogenic differentiation of stretch-induced PDLSCs, Wei’s group tested the correlation analysis between stretch times and miR-21 [46,51]. The expression of miR-21 and osteogenic marker genes (*Runx2* (runt related transcription factor 2) and *Ocn*) gradually rose with stretch times increasing by degrees. Further, miR-21’s target gene *Acvr2b* (activin receptor type IIB) partially inhibited the stretch-induced ALP activity and *Runx2* and *Ocn* gene expression, revealing that mechanosensitive miR-21 facilitated stretch-induced PDLSC osteogenic differentiation through suppressing Acvr2b. The miR-21 level was down-regulated approximately 70% under FSS in MC3T3-E1 cells [48]. Additionally, miR-21 was reported to promote the level of osteogenic differentiation and increase matrix mineralization by directly repressing Smad7 in MC3T3-E1 cells [52], while miR-21 expression gradually decreased during PDLC mineralization [53]. The results of these studies indicated that miR-21 might have a diametric role in different types of bone cell osteogenic differentiation and under various mechanical stimuli. This phenomenon also appeared in another miRNA (miR-33), which was reported to decrease in the mechanical stretch group in MC3T3-E1 cells [41]. However, one subtype of miR-33, miR-33-5p expression, was significantly up-regulated under increasing FSS, and the knockdown of miR-33-5p partially prevented FSS-induced osteoblast differentiation [40]. Not only was miR-33-5p sensitive to mechanical loading, but it was also responsive to mechanical unloading in MC3T3-E1 cells. In view of miR-33-5p positively regulating osteoblast differentiation, Wang investigated the roles of miR-33-5p in osteoblast activity under mechanical unloading. The overexpression of miR-33-5p partially attenuated the inhibition of MC3T3-E1 osteogenic differentiation induced by mechanical unloading and *Hmga2* (high mobility group AT-hook 2), as miR-33-5p’s target, was confirmed to negatively regulate osteoblast differentiation [40].

FSS was also used to study the effects of mechanical stimuli on PDLCs. It is reported that increasing FSS in fixed increments regulated not only the PDLC proliferation and differentiation, but also miR-132 expression. Further, phosphorylated levels of the PI3K (phosphatidylinositol-3 kinase), AKT (serine/threonine kinase), mTOR (mammalian target of rapamycin), and p70S6K (ribosomal protein S6 kinase) proteins were significantly greater with FSS-treatment, however they were nonetheless blocked by the miR-132 inhibitor. The subsequent results suggested that up-regulation of miR-132 induced by FSS could activate the mTOR signaling pathway and PDLC osteogenic differentiation [39]. Bio-stretch was another type of stretch stress device, which exerted a uniaxial stretch with square wave patterns and was used to simulate stretch in chondrocytes. miR-365 was identified as the first mechanosensitive miRNA to stimulate chondrocyte differentiation via directly targeting *Hadc4* (hystone deacetylase 4) [38].

### 3.2. Mechanosensitive miRNAs Inhibited Osteogenic Differentiation

Despite several mechanosensitive miRNAs promoting osteogenic differentiation (Table 2), there are more mechanosensitive miRNAs reported to serve as inhibitors in osteogenic differentiation (Table 3). Li et al. have demonstrated miR-154-5p was remarkably down-regulated in adipose-derived mesenchymal stem cells (ADSCs) subjected to four-point bending stretch. ALP activity and matrix mineralization were altered treatments with lentivirus-miR-154-5p or inhibitor of miR-154-5p under mechanical stretch, which indicated that mechanical-stretch-sensitive miR-154-5p prevented osteogenic differentiation of ADSCs through the Wnt/PCP (planar-cell-polarity) pathway by directly targeting *Wnt11* (Wnt family member 11) [37]. Another miR-195-5p also targeted a Wnt family member (*WNT 3A*) to inhibit PDLC differentiation under Flexcell stretch. In addition, miR-195-5p was involved in FGF (fibroblast growth factor) and BMP signaling through targeting *FGF2* and *BMPR1A* (bone morphogenetic protein receptor-1A) during osteogenic differentiation of PDLCs [54]. Although compression force was a type of mechanical loading condition, the effects of compressive force on osteogenic differentiation were opposite to other loading methods [55]. It is reported that the miR-29 family, including miR-29a, miR-29b and miR-29c, significantly decreased approximately 0.5-fold and suppressed the target extracellular matrix gene (*Col1a1*, *Col3a1* and *Col5a1*) expression in PDLCs treated with Flexcell stretch. On the contrary, the significantly increased (1.8–4-folds) expression of the miR-29 family were detected when PDLCs were subjected to compressive force [55].

In addition to regulating PDLSCs and PDLCs osteogenic differentiation, Flexcell-strain-sensitive miRNA contributed to osteoblast and Bone marrow mesenchymal stem cells (BMSC) osteogenic differentiation [22,23]. Zuo et al. identified that a mechanosensitive miRNA, miR-103a, reduced osteoblast activity and matrix mineralization through binding to *Runx2* 3′UTR in hFOB 1.19 cells under Flexcell stretch [23]. Recently, miR-503-5p was observed to be a negative regulator of stretch-mediated osteogenic differentiation in BMSCs [22].

Extracorporeal shockwave (ESW), another type of mechanical stress, transduced the mechanical signals into biological signals and promoted osteogenic differentiation [56]. The miR-138 level was decreased with post-ESW treatment and the overexpression of miR-138 abrogated osteogenic differentiation induced by ESW through down-regulating *FAK* (Focal adhesion kinase), *Runx2* expression and mineralization in MSCs (mesenchymal stem cells) [57]. In BMSCs, miR-138 was reported to inhibit osteogenic differentiation by targeting FAK, and antagomir-138 (miR-138 inhibitor) obviously enhanced ectopic bone formation [58,59].

In a preceding study, miR-132 positively regulated FSS-induced PDLC osteogenic differentiation through the mTOR signaling pathway [39]. However, miR-132-3p, one subtype of miR-132, was up-regulated (about 2.5-fold) in a mechanical unloading group and negatively correlated with osteoblast differentiation in primary rat osteoblasts. Moreover, *Ep300* (E1A binding protein p300), miR-132-3p’s target, significantly decreased under mechanical unloading and suppressed the activity and acetylation of Runx2, a key regulatory factor of osteoblast differentiation [60]. This is further evidence that the same miRNA played opposite roles in the differentiation of different osteogenic cells subjected to various mechanical stimuli. Mechanosensitive miRNAs temporally changed with mechanical use/disuse, and played important roles in osteogenic differentiation under different mechanical conditions. Without mechanical stimuli, the gain or loss of function of the mechanosensitive microRNAs still affects many bio-active molecules indispensable to the regulation of osteogenic differentiation in nature [21,61,62,63].

## 4. Mechanosensitive miRNAs and Osteoblast Proliferation

Not only did osteoblast differentiation change, but osteoblast proliferation also altered with the transformation of mechanical stimuli (Table 4). Experimental evidence showed that osteoblasts under compressive force at 294 Pa had slower cell growth and higher expression of miR-494-3p compared with no-compressive group cells. Moreover, overexpression of miR-494-3p repressed osteoblast proliferation assay through targeting *Fgfr2* (fibroblast growth factor receptor 2) and *Rock1* (Rho associated coiled-coil containing protein kinase 1) [36]. Zhang’s group paid massive attention to the effects of mechanical unloading on osteoblast activity, and they found that a mechanical unloading of sensitive miRNA, miR-103 [64,65], was involved in osteoblast proliferation. Increasing miR-103 expression and a decreasing number of EdU (5-Ethynyl-2′-deoxyuridine) positive cells induced by mechanical unloading demonstrated that miR-103 was negatively correlated to MC3T3-E1 cell proliferation. The miR-103’ target *Cav1.2* (calcium voltage-gated channel), an L-type voltage-sensitive calcium channel (LTCC), decreased under the mechanical unloading environment, which was moderated by the miR-103 inhibitor [64,65]. Taken together, the roles of mechanosensitive miRNAs in bone cells were concentrated on osteogenic differentiation and osteoblast proliferation.

## 5. Mechanosensitive miRNAs and Bone Formation

It is reported that some mechanosensitive miRNAs are involved in bone formation in vivo (Table 5). Accumulating evidence suggests that almost 40% of reports of exercise-sensitive miRNAs were in relation to skeletal muscle [66], but less to bone in vivo. Exercise, a natural method to investigate the influence of mechanical loading on bone formation in vivo, is well known to strongly benefit trabecular bone parameters, bone mineral density (BMD) and bone mechanical strength, including treadmill walking, swimming and artificial cyclic strain (four-point bending on tibia) [67,68,69]. Daily bouts of exercise [70] or high-frequency motions [71] were seen as the primary interventions to reduce the risk of bone loss. Conditional knockout (cKO) miR17-92 cluster in collagen type I-producing osteoblasts resulted in a 13–34% reduction of total body bone mineral content, a 10% reduction in maximum load, a 28% reduction in the periosteal bone formation rate, but no change in bone toughness and the resorbing surface [72]. Interestingly, miR17-92 cluster cKO mice subjected to two weeks of mechanical exercise by four-point bending, revealed no obvious change in tibia periosteal bone formation, compared to wild-type mice [72]. Besides, Sengul reported that miR-92 inhibitor (antagomir-92) increased tibia TV (Tissue volume), BV/TV (Bone volume/tissue volume) and density by 7–16% after two weeks mechanical exercise, but a similar increase in the above parameters (6–15%) was seen in the antagomir-NC (inhibitor negative control) treated group with two weeks mechanical exercise [73]. Both studies revealed that the mechanosensitive miR17-92 cluster blocked bone formation in response to mechanical exercise loading. miR-222, a potential regulator of an articular cartilage mechanotransduction pathway, increased in the anterior weight-bearing area of articular cartilage as compared with the posterior non-weight-bearing area with minimum contact pressure [74].

Apart from weight-bearing bone formation, alveolar bone formation was also influenced by mechanosensitive miRNAs under mechanical loading. As a matter of fact, alveolar bone formation and incisor tooth mechanical properties also decreased in miR17-92 cluster cKO mice [75]. The role of mechanosensitive miR-21 in PDLSCs has been investigated in vitro [51]. Similarly, miR-21 was dose- and time-responsive to orthodontic force, which induced alveolar bone formation in vivo [76]. During orthodontic tooth movement (OTM), miR-21 deficiency prevented force-induced alveolar bone formation just in the tensile side, while blocking bone resorption in both the compressive and tensile sides in miR-21 knockout mice [76]. Meanwhile, Liu, et al. indicated that miR-503-5p was involved in OTM and the overexpression of miR-503-5p reduced *Runx2*, *Alp* mRNA and protein expression, and decreased osteoblast numbers and osteoblastic bone formation in the OTM tension sides [22].

While a lack of mechanical loading or physical activity disturbed the delicate balance of the homeostasis of weight-bearing bones [77], and a reduction in bone formation is frequently observed in the aging and in paraplegics [78]. It was known that sense and response to mechanical stimuli in middle-aged and old mice was less than in young adults, which led to the aged skeleton diminishing in bone formation [14,79,80]. There is growing evidence that age-altered miRNAs modulated bone formation [81,82,83]. More recently, miR-188 was identified as a key regulator of the age-associated switch between osteoblast and adipocyte differentiation of BMSCs [84]. Notably, the miR-188 level was positively correlated with age in both mouse and human BMSCs, whereas it was negatively correlated with trabecular bone formation parameters (BV/TV, Tb.N (Trabecular number)) and tibia maximum mechanical load. With aging, miR-188 knockdown in animals reduced decrease rates of bone formation and tibia mechanical strength; conversely, miR-188 overexpression in transgenic mice revealed a substantial age-related reduction of bone formation and tibia maximum mechanical load. From the molecular mechanism point of view, miR-188 inhibited the osteogenic differentiation of BMSCs by targeting hystone deacetylase 9 (*Hdac9*). In addition, the miR-188 inhibitor—via a BMSC-specific aptamer—relieved age-induced osteoporosis and reduced femur maximum mechanical load [84]. It is reported that miR-214 [85] was up-regulated, while miR-142-5p [86], miR-31 [83], the miR 17-92 cluster [82], miR-let-7f, miR-125b, miR-199-3p and miR-222 [87] were down-regulated with age increasing in different osteogenic cells or bone tissue in vivo.

In addition, external mechanical unloading resulted in the deterioration of bone micro-structure, particularly the trabecular bone compartment and weak mechanical properties in vivo [88,89]. Although the mechanosensitive miR-103 family has been identified as negatively regulating osteoblast differentiation [23] and proliferation [64,65] in vitro, there is still conflicting evidence showing either stimulatory or inhibitory effects of the miR-103 family on bone formation under mechanical unloading in vivo [23,90]. Pretreatment with miR-103a inhibitor partly counteracted the decrease of bone formation caused by hind-limb unloading [23], nevertheless the expression of miR-103-3p (same with miR-30b-5p and miR-142-3p) was significantly declined in the serum of rhesus monkeys after long-duration bed-rest and positively correlated with BMD [90]. Bed-rest was a reliable model for the simulation of mechanical unloading and miRNAs, as putative circulating biomarkers of diseases, were stable in plasma and serum. Li’s group also demonstrated the circulating miRNA profile of plasma from 16 individuals after 45 days of −6° head-down tilt bed-rest. 11 miRNAs including miR-103, -130a, -1234, -1290, -151-5p, -151-3p, -148a, -199a-3p, -20a, -363, and -451a revealed dramatically decreased after 45 days of bed-rest, while the expressions of these miRNAs were recovered after 10 days of mechanical recovery, except miR-148a, -199a-3p and -151-3p. Additionally, several miRNAs, especially miR-1234, were identified in positive correlation with bone formation parameters [91]. Expressions of miR-214 [85], miR-139-3p, miR-339-3p, miR-132-3p, miR-487b, miR-2985 and miR-34b [60] have been demonstrated to alter in the bones of hind-limb unloading animals. Significantly, miR-214 level was reported to vary with natural age in human osteoporotic bone specimens and increase in Alp^+^ cells isolated from bones after 28 days hind-limb unloading, which implied that miR-214 might be potentially sensitive to mechanical unloading and played a crucial role in bone formation [85]. Furthermore, miR-214 has been shown to repress osteogenic differentiation or bone formation in different osteogenic cells through binding to various protein coding genes, such as *Osterix* in C2C12 myoblast cells [92], activating transcription factor 4 (*ATF4*) in osteoblasts [85], and *FGF* in BMSCs [93]. The roles of mechanosensitive miRNAs in bone formation were showed in Table 5.

## 6. Conclusions and Perspectives

An increasing number of studies are showing that some miRNAs (e.g., miR 17-92 cluster, miR-103 family) have been identified in response to different mechanical stimuli and to act as crucial regulators in osteogenic differentiation and bone formation. However, controversial functions of mechanosensitive miRNAs existed in osteogenic differentiation under different mechanical stimuli: (1) Stretch stress and FSS showed the opposite effects of mechanosensitive miRNAs on osteogenic differentiation, for instance miR-21 and miR-33; (2) The expression of the mechanosensitive miR-132 family showed the same trend under opposite mechanical stimuli.

The future perspectives for the application of mechanosensitive miRNAs may include: (1) several mechanosensitive miRNAs mimics or inhibitors potentially served as therapeutic approaches for osteopenia due to a lack of mechanical loading; (2) the potential application of mechanosensitive miRNAs as biomarkers to assess the extent of osteopenia induced by the alteration of mechanical stimuli in patients’ serum, plasma or exosomes; (3) exercise-sensitive miRNAs presumably accounted for bone-muscle interactions, owing to exercise changing many miRNAs expression in skeletal muscles; (4) mechano-sensitive miRNAs related to bone resorption is a compelling future research direction, since osteoclastogenesis and osteoclast activity altered with different mechanical stimuli [94,95,96]. Despite the functional roles of mechanosensitive miRNAs in osteogenic differentiation and bone formation being elucidated in the present review, the mechanisms of miRNA response to different mechanical stimuli during bone remodeling and clinical practice remain to be investigated in future.

## Figures and Tables

**Table 1:** Mechanosensitive miRNAs screened by microarray are putatively involved in osteogenic differentiation.

miRNAs	Mechanical Stimuli and Parameters	Microarray Groups	Samples	Reference
miR-218, -33, 191*, 3070a	Stretch stress (Four-point bending) 2500 µε, 0.5 Hz	0 and 8 h	MC3T3-E1	[41]
miR-3077-5p, -3090-5p, -3103-5p, -466h(i)-3p	Stretch stress (Four-point bending) 2500 με, 0.5 Hz	0 and over 3 d (1 h/d)	MC3T3-E1	[42]
miR-146b-5p	Stretch stress (Four-point bending) 2000 µε, 0.5 Hz	0 and 18 h	OCCM-30	[43]
miR-1246, -5096, -638, -663, -21, -4492, -4734, -3195, -4281, -3178	Stretch stress (Flexcell) 10% strain, 1.0 Hz	0 and 12 h	PDLSCs	[46]
miR-195-5p, -424-5p, -1297, -3607-5p, -145-5p, -4328, 224-5p	Stretch stress (Flexcell) 12% deformation 0.1 Hz	0 and 72 h	PDLCs	[47]
miR-20a, -21, 199b, -34a(c), -140, -200b	FSS 12 dyn/cm^2^	0, 0.5 and 1 h	MC3T3-E1	[48]

d: day; FSS: Fluid shear stress; h: hour; miR: microRNA; OCCM: immortalized osteocalcin positive cementoblasts; PDLCs: periodontal ligament cells; PDLSCs: Periodontal ligament stem cells.

**Table 2:** Mechanosensitive miRNAs promote osteogenic differentiation.

miRNAs	Mechanical Stimuli	Methods	Target Gene	Samples	Reference
miR-21	Stretch stress (Flexcell)	10% strain 1.0 Hz 6, 12, 24, or 48 h	*Acvr2b*	PDLSCs	[51]
miR-365	Stretch stress (Bio-stretch)	8% deformation, 1 Hz 15 min/h, 24 h	*Hdac4*	chondrocyte	[38]
miR-132	FSS	3, 6, 9, 12, 15 dyn/cm^2^, 6 h	*-*	PDLCs	[39]
miR-33-5p	FSS Unloading	10 dyn/cm^2^, 1 h 24 rpm, 48 h	*Hmga2*	MC3T3-E1	[40]

*Acvr2b*: activin receptor type IIB; FSS: Fluid shear stress; *Hmga2*: high mobility group AT-hook 2; *Hdac4*: hystone deacetylase 4; PDLCs: periodontal ligament cells; PDLSCs: Periodontal ligament stem cells.

**Table 3:** Mechanosensitive miRNAs inhibit osteogenic differentiation.

miRNAs	Mechanical Stimuli	Methods	Target Gene	Samples	Reference
miR-154-5p	Stretch stress (Four-point bending)	2000 με, 0.5 Hz 2 h/day	*Wnt 11*	ADSCs	[37]
miR-103a	Stretch stress (Flexcell)	8% elongation, 0.5 Hz, 72 h	*Runx2*	hFOB1.19	[23]
miR-195-5p	Stretch stress (Flexcell)	12% deformation 0.1 Hz, 72 h	*WNT3A BMPR1A* *FGF2*	PDLCs	[54]
miR-503-5p	Stretch stress (Flexcell)	10% elongation, 1 Hz, 12 h	*-*	BMSCs	[22]
miR-29 family (a,b,c)	Stretch stress (Flexcell) Compressive force	12% deformation, 0.1 Hz, 24 h, 2 g/cm^2^, 24 h	*Col1a1 Col3a1 Col5a1*	PDLCs	[55]
miR-138	Shockwave (ESW)	0.16 mJ/mm^2^, 500 impulses, 10 min	*FAK*	MSCs	[57]
miR-132-3p	Unloading	30 rpm, 48 h	*Ep300*	primary osteoblast	[60]

ADSCs: adipose-derived mesenchymal stem cells; *BMPR1A*: bone morphogenetic protein receptor-1A; BMSCs: Bone marrow mesenchymal stem cells; *Col I*: Collage I; *Ep300*: E1A binding protein p300; ESW: Extracorporeal shockwave; *FAK*: Focal adhesion kinase; *FGF2*: fibroblast growth factor 2; FSS: Fluid shear stress; *Hmga2*: high mobility group AT-hook 2; miR: microRNA; MSCs: mesenchymal stem cells; PDLCs: periodontal ligament cells; *Runx2*: runt related transcription factor 2; *WNT3A*: WNT family member 3A. *WNT11*: WNT family member 11.

**Table 4:** Mechanosensitive miRNAs inhibit osteoblast proliferation.

miRNAs	Mechanical Stimuli	Methods	Target Gene	Samples	Reference
miR-494-3p	Compressive force	294 Pa (2.0–4.0 g/cm^2^), 24 h	*Fgfr2*, *Rock1*	MC3T3-E1	[36]
miR-103	Unloading	24 rpm, 48 h	*Cav1.2*	MC3T3-E1	[64,65]

*Cav1.2*: calcium voltage-gated channel; *Fgfr2*: fibroblast growth factor receptor 2; miR: microRNA; *Rock1*: Rho associated coiled-coil containing protein kinase 1; rpm: revolutions per minute.

**Table 5:** The roles of mechanosensitive miRNAs in bone formation.

miRNAs	Mechanical Methods	Source	Supporting Observations	Reference
miR17-92 cluster	four-point bending device at 2 Hz for 36 cycles for 2 weeks	Osteoblast cKO mice	Block tibia periosteal bone formation response to mechanical exercise loading in the cKO mice; alveolar bone size and incisor tooth mechanical properties decreased in the cKO mice.	[72,73,75]
miR-222	anterior weight-bearing and posterior non–weight-bearing regions	bovine articular cartilage	A potential regulator of an articular cartilage mechanotransduction pathway.	[74]
miR-21	orthodontic force in periodontal tissue at 30 g of force for 7 d	miR-21−/− mice	miR-21 deficiency prevented force-induced alveolar bone formation just in the tensile side, while blocking bone resorption in both the compressive and tensile sides.	[76]
miR-503-5p	the left maxillary first molars were mesially stretched at 50 g of force for 1, 3, 7, 14 d respectively	alveolar bones	Agomir-503-5p decreased *Runx2*, *Alp* mRNA and protein expression, decreased osteoblast numbers and osteoblastic bone formation in the orthodontic tooth movement (OTM) tension sides.	[22]
miR-188	3, 6, 12, 18-month mice 85 human females and 85 males at different ages	BMSCs	miR-188 level increased with age. Further, miR-188 deficiency revealed substantially reduced decrease of age-associated rates of bone formation and tibia maximum mechanical load; miR-188 transgenic mice determined opposite results; miR-188 inhibitor via a BMSC-specific aptamer relieved age-induced osteoporosis and reduction of femur maximum mechanical load.	[84]
miR-214	20 human females and 20 males at different ages	Bone specimens	Expression of miR-214 increased with age and was negatively correlated with the expressions of bone formation marker genes.	[85]
miR-142-5p	Mice at 10 weeks and 18 months of age	Bone callus	miR-142-5p level was lower in the callus of aged mice than the young group, and Agomir-142-5p repaired the aged bone fractures.	[86]
miR-31	Mice at 2, 8, 25 months of age	MSCs and bone tissue	miR-31 level showed a consistent decreased trend with age increasing in MSCs and bone.	[83]
**miRNAs**	**Mechanical Methods**	**Source**	**Functions**	**Reference**
miR17-92 cluster	Mice at 4 and16 months of age	BMSCs and bone tissue	miR-17 mimics could partially rescue the osteogenesis of old MSCs both in vitro an in vivo	[82]
miR-let-7f, -199-3p, -125b, -222	22 healthy rhesus macaques young (<5 years), middle (8–10 years), old (>12 years, *n* = 9)	BMSCs	Analysis of miRNA expression profiles and qPCR results revealed a down-regulation of miR-let-7f, miR-125b, miR-222 and miR-199-3p in old rBMSCs.	[87]
miR-103-3p, -30b-5p, -142-3p	10-degree head-down tilt position for 42-day long-duration bedrest	serum of rhesus monkeys	Expressions of the three miRNAs declined in the serum of rhesus monkeys after bedrest and positively correlated with BMD.	[90]
miR-103, -130a, -1234, -1290, -151-5p, -151-3p, -199a-3p, -20a, -148a, -363, -451a	16 male volunteers after 45 days of −6° head-down tilt bedrest	plasma of individuals	These miRNAs were dramatically decreased after 45 days of bedrest, while the expressions of these miRNAs were recovered after 10 days of recovery, except miR-148a, 199a-3p and 151-3p. Additionally, several miRNAs especially miR-1234 was identified in positive correlation with bone formation parameters.	[91]
miR-214	Hind-limb unloading for 28 days	*Alp*^+^ cells and bone tissue	miR-214 level was up-regulated after 28-day hind-limb unloading, and Antagomir-214 counteracts unloading induced bone loss.	[85]
miR-103a	Hind-limb unloading for 28 days	bone tissue	Antagomir-103a partly resumed unloading-caused bone loss.	[23]
miR-139-3p, -339-3p, -132-3p, -487b, -2985, -34b	Hind-limb unloading for 21 days	bone tissue	the expression levels of miR-139-3p, -339-3p and -132-3p were up-regulated, and the expression levels of miR-487b, -2985 and -34b were down-regulated in rat bone after three weeks tail suspension.	[60]

Alp: alkaline phosphatase; Alp^+^: Alp positive; BMD: bone mineral density; BMSCs: bone marrow mesenchymal stem cells; cKO: conditional knockout; d: days; miR: microRNA; MSCs: mesenchymal stem cells; OTM: Orthodontic tooth movement; Runx2: Runt related transcription factor 2.

## Abbreviations

Acvr2b	Activin receptor type IIB
ADSCs	Adipose-derived mesenchymal stem cells
Ago2	Argonaute 2
AKT	Serine/threonine kinase
ALP	Alkaline phosphatase
Alp^+^	Alp positive
ATF4	Activating transcription factor 4
BFR	Bone formation rate
BMD	Bone mineral density
BMMs	Bone marrow monocytes
BMPR1A	Bone morphogenetic protein receptor-1A
BMSCs	Bone marrow mesenchymal stem cells
BMP	Bone morphogenetic protein
Bsp	Bone sialoprotein
BV/TV	Bone volume/tissue volume
cKO	Conditional knockout
Col I	Collage I
Cav1.2	Calcium voltage-gated channel
Creb1	CAMP responsive element binding protein 1
DGCR8	Digeorge syndrome critical region gene 8
Ep300	E1A binding protein p300
EdU	5-Ethynyl-2′-deoxyuridine
ESW	Extracorporeal shockwave
FAK	Focal adhesion kinase
FGF	Fibroblast growth factor
Fgf2	Fibroblast growth factor 2
Fgfr2	Fibroblast growth factor receptor 2
FSS	Fluid shear stress
Hmga2	High mobility group AT-hook 2
Hdac4	Hystone deacetylase 4
Hdac9	Hystone deacetylase 9
LTCC	L-type voltage-sensitive calcium channel
MAR	Mineral apposition rate
mTOR	Mammalian target of rapamycin
MSCs	Mesenchymal stem cells
miRNAs	MicroRNA
OCCM	Immortalized osteocalcin positive cementoblasts
mRNAs	Messenger RNAs
Ocn	Osteocalcin
OTM	Orthodontic tooth movement
PCP	Planar-cell-polarity
PDLCs	Periodontal ligament cells
PDLSCs	Periodontal ligament stem cells
PI3K	Phosphatidylinositol-3 kinase
P70S6K	Ribosomal protein S6 kinase
pri-miRNA	Primary transcript
pre-miRNA	Precursor miRNA
Pten	Phosphatase and tensin homolog
RISC	RNA-Induced Silencing Complex
Rock1	Rho associated coiled-coil containing protein kinase 1
rpm	Revolutions per minute
RPM	Random positioning machine
Runx2	Runt related transcription factor 2
RWVB	Rotating Wall Vessel Bioreactor
Smad4	SMAD family member 4
Tb.N	Trabecular number
TV	Tissue volume
TGF-β	Transforming growth factor-β
UTR	Untranslated region
WNT3A	WNT family member 3A
WNT 11	WNT family member 11
